# CDFT-Based Reactivity Descriptors as a Useful MEDT Chemoinformatics Tool for the Study of the Virotoxin Family of Fungal Peptides

**DOI:** 10.3390/molecules24152707

**Published:** 2019-07-25

**Authors:** Norma Flores-Holguín, Juan Frau, Daniel Glossman-Mitnik

**Affiliations:** 1 Laboratorio Virtual NANOCOSMOS, Departamento de Medio Ambiente y Energía, Centro de Investigación en Materiales Avanzados, Miguel de Cervantes 120, Complejo Industrial Chihuahua, Chihuahua, Chih 31136, Mexico; 2 Departament de Química, Universitat de les Illes Balears, 07122 Palma de Mallorca, Spain

**Keywords:** virotoxins, chemical reactivity theory, conceptual DFT, pKa, chemoinformatics, computational peptidology

## Abstract

Virotoxins are monocyclic peptides formed by at least five different compounds: alaviroidin, viroisin, deoxoviroisin, viroidin and deoxovirodin. These are toxic peptides singularly found in Amanita virosa mushrooms. Here we perform computational studies on the structural and electronic conformations of these peptides using the MN12SX/Def2TZVP/H2O chemistry model to investigate their chemical reactivity. CDFT-based descriptors (for Conceptual Density Functional Theory) (e.g., Parr functions and Nucleophilicity) are also considered. At the same time, other properties (e.g., pKas) will be determined and used to study virotoxins solubility and to inform decisions about repurposing these agents in medicinal chemistry.

## 1. Introduction

Rapid advancement in medical technology has made it possible to develop highly effective tools and models to enhance toxicology and pharmacological processes. These tools and techniques significantly enhance the application of technology in the improvement of the methods used in the risk assessment of drugs and also determine their levels of efficiency. Moreover, the development of new computational tools and techniques in the pharmacological and toxicological processes potentially increases the efficiency of experimental designs thus minimizing the number of trials required to establish the efficacy of a drug. Fields such as Computational Peptidology are rapidly advancing to promote the integration of multidimensional data in the development of advanced prediction models to assess the potential effects of certain chemicals on living organisms or the environment. Advancement in the computational toxicology involves the integration of different biochemistry, medicine, mathematics, and engineering concepts to determine how different biological organisms react with chemical agents at the molecular, group, individual, and cellular levels. It is also applied in different safety screening and risk prioritization processes to determine the drug metabolites. As a result, different government agencies have, private organizations, and learning institution has developed computational techniques to advance this field of knowledge and also create new innovations [1].

The study of chemoinformatics began half a century ago as researchers conducted clinical trials to determine how molecular sub-structural patterns influence the biological activity in organisms. This field of study has evolved over the years to include the development of advanced tools and techniques in the discovery of the underlying commercial value of different molecular structures. This explains the definition of chemoinformatics as the study of computational methods involved in the process of design, organization, analysis, and dissemination of chemical data to inform the discovery of new medical drugs, agrochemicals, and the food industry. Every innovation whose background is in chemoinformatics focuses on the analysis of chemical structures and bioreactivity properties of molecular structures through various chemical coupling and computational modeling. One of the most important aspects of integrating biological properties with chemical reactivity of molecular structures is the application in the modification of molecular descriptors [2].

Proteins have been recognized as the life building blocks although advancement in the biochemical technology has led to the discovery of vital elements known as peptides that are found in essentially all life processes. Biological trials have revealed that active peptides are found in important body organs such as the heart and brain. Additionally, researchers have found compounds containing peptide properties in a wide range of antibiotics and toxins. Natural processes have been responsible for the integration of diverse amino acids to produce a wide range of complex agonists. Being an independent field of study, it is important that peptide chemistry operates as a major branch of biochemistry without being integrated with other chemistry proteins, as it plays a key role in the study of organic chemistry [3].

Toxicology significantly influences important aspects of our day to day lives either knowingly or obliviously, especially due to the application in the safety characterization of different consumer products. Of all 10,000 mushroom species that have been discovered, about 250 species are known to have negative effects on the human body when consumed, while some species contain fatal toxins [4]. Phallotoxin and amatoxins are valuable peptides which are known to contain a toxin that is fatal for human consumption while virotoxins also contain less harmful toxins. Virotoxins, phallotoxins, and amatoxins are known to contain Penta monocyclic heptapeptides [5]. The relationship between these virotoxins and phallotoxins is based on their level of toxicity and molecular structures although they do not result in acute toxicity when ingested [6]. All categories of virotoxins are said to be AWLATCP amino acid variants based on the assumption that they are derivatives of phallotoxin-like molecules. The inexistence of trypthionine cross-bridge in the phallotoxins give the virotoxins monocyclic properties. Therefore, amino acid category one contains Ala, category two is usually L-Trp modified with methysulfinyl and methylsulfonyl, category three is usually L-Leu hydroxylated with phallotoxin-like carbon groups, category four is in most cases L-Ala unmodified just like the phallaidin, and the phallotoxins in amino acid category six are made of D-Ser instead of L-Cys. Amino acids category 7 contains 3,4-dihydroxy-L-Pro properties since it is stereochemically, 3-trans, 3,4 trans isomer considering the virotoxin nomenclature, especially those whose din end contains dihydroxylated Leu number 3 and those whose sin end contain trihydroxylated properties [7,8].

According to the Chemical Reactivity Theory [9,10], series descriptors are vital in the analysis of the AGE inhibitors based on their molecular properties and the initial point of development of the new inhibitors. The analysis of descriptors is based on their respective neutral system energies and the application of the ΔSCF method in the computation of neutral system energies. This is based on the variation in the systematic energies between the radical ions and the neutral ions. The computation process can be simplified by the application of the KID (for Koopmans in DFT) method based on the identification of their vertical electron affinity and the ionization potential with HOMO energy. The KID technique is considered an approximation of the energy values since the application of Koopmans’ theorem is not valid within the DFT [11,12,13,14]. However, this method is reliable in the faster computation of conceptual DFT descriptors within large molecular systems given that the determination of analysis of the anions and cations based on their electronic energy is faced by convergence and cost challenges.

According to the Molecular Electron Density Theory (MEDT) proposed by Luis R. Domingo [15] on the reactivity patterns in organic chemistry, the probability of change in the electron density of a molecule significantly affects its molecular reactivity while the physical and chemical properties are influenced by the electron density distribution [15]. A wide range of reactivity descriptors and concepts have been used to explain the conceptual DFT based on the Nucleophilicity index, the global electronic density transfer, and the local condensed descriptors such as Parr functions (nucleophilic P${}_{k}^{+}$ and electrophilic P${}_{k}^{-}$) [16]. Our recent publications on the reactivity properties of different bioactive peptides focused on the evaluation of the MN12SX/DefTZVP/H2O model effectiveness in the computation of the chemical reactivity of at least 5 classes of the virotoxin family of peptides containing fungal structures. In this case, the analysis is based on the computation of the global descriptors within the KID technique and the conceptual DFT. Therefore, it is important to note that we intend to predict the chemical reactivity patterns of the CDFT descriptors subject to the Parr functions and not to compute the vertical I and A through a comparative analysis with the experimental results.

As a follow-up of our recently published work on the chemical reactivity properties of potentially bioactive peptides [17,18,19,20,21,22], the objective of this work is to perform a comparative study of the performance of the MN12SX/DefTZVP/H2O model chemistry for the prediction of the chemical reactivity of the five members of the Virotoxin family of fungal peptide and to proceed to the verification of the mentioned KID procedure. It must be stressed that there is not our intention to calculate the vertical I and A to compare them to experimental results, but to predict trends of chemical reactivity on the basis of those CDFT descriptors as well as the considerations of the Parr functions.

## 2. Computational Methodology

Following the methodology considered in our previous works [17,23,24,25,26,27,28,29], similar computations have been performed in this work by resorting to the Gaussian 09 software [30]. The full methodological procedure is explained in detail at the beginning of the Results and Discussion section. In a similar way as it was done in the referenced works, the MN12SX density functional [31] was considered due to the fact that it is a well-behaved density functional for our purposes according to our proposed KID (for Koopmans in DFT) criteria [17,23,24,25,26,27,28,29]. related to the approximate validity of the Koopmans’ theorem within DFT [32,33,34,35,36]. For the calculation of the electronic properties a model chemistry has been considered on the basis of the MN12SX density functional associated with the Def2TZVP basis set, while a smaller Def2SVP was considered for the prediction of the most stable structures [37,38]. All calculations were performed using water, which is the universal biological solvent, simulated with the SMD model [39].

## 3. Results and Discussion

**Figure 1:**
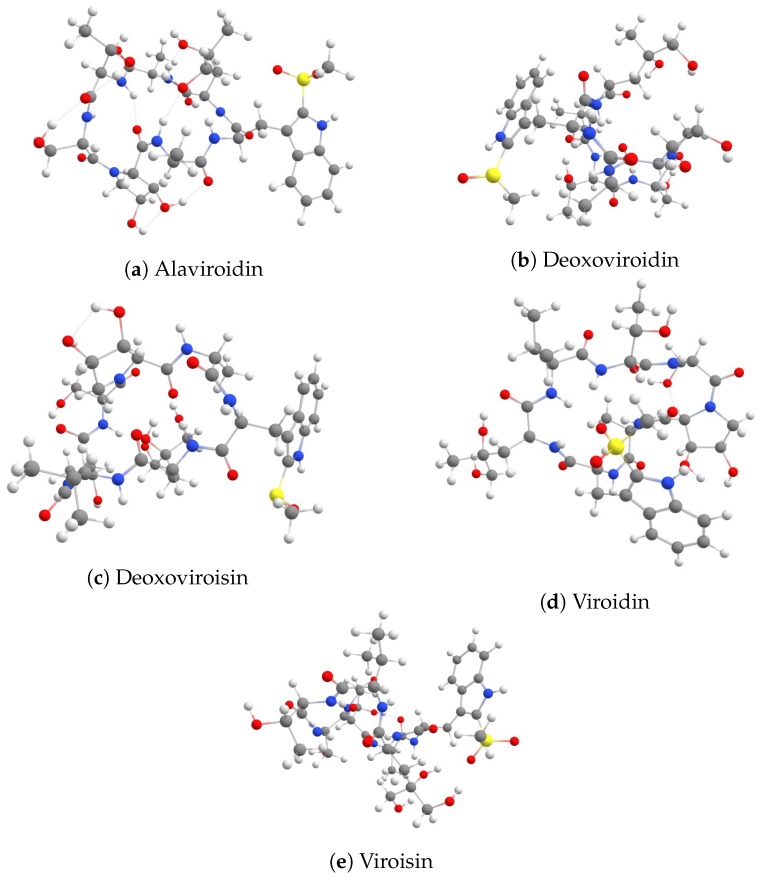
Graphical sketches of the molecular structures of the five members of the Virotoxin family of peptides.

The molecular structures of the Virotoxin family of peptides drawn by scratch are depicted in Figure 1, were optimized in gas phase by resorting to the DFTBA model, through the consideration of the five most stable conformers chosen from a pre-optimization accomplished by means of Molecular Mechanics techniques [40,41,42,43,44] using the conformers searching engine available in the Marvin View 17.15 program, which can be regarded as an advanced chemical viewer (https://www.chemaxon.com). All the resulting conformers were processed, as is customary within Computational Chemistry, by means of a new reoptimization with the MN12SX density functional mentioned before together with the Def2SVP basis set and the SMD solvent model, using water as the solvent. Once it has been verified that every structure belonged to the minimum energy conformation by means of a frequency calculation analysis, the corresponding electronic properties were calculated with the Def2TZVP basis set instead of that used for the geometry optimization. To obtain self-convergence and to avoid the propagation of errors we have considered in this study the tight convergence criteria available in Gaussian 09 as well as an ultrafine grid for the calculation of the required integrals.

As it has been mentioned recently by Becke [45] and also by Baerends et al. [46], it can be said that the lowest excitation energy can be associated with the HOMO–LUMO gap of the ground state [47]. Therefore, in this work, the determination of the maximum wavelength absorption of the five peptides of the Virotoxin family was done by conducting ground-state calculations with the aforementioned density functional at the same level of model chemistry and theory and then determining the HOMO–LUMO gaps from which the maximum absorption wavelengths $\lambda_{max}$ were obtained (Table 1).

### 3.1. Calculation of Global Reactivity Descriptors

According to the results obtained when studying melanoidins [23,24,25,26,27,28,29] as well as peptides from marine sources [17], it can be said that the calculations performed with the MN12SX density functional render HOMO and LUMO energies that satisfy the approximate Koopmans’ theorem. Thus, the application of the KID procedure will be justified. The global reactivity descriptors Electronegativity χ [9,48], Global Hardness η [9,48], Electrophilicity ω [49], Electrodonating Power $\omega^{-}$ [50], Electroaccepting Power $\omega^{+}$ [50] and Net Electrophilicity $\Delta \omega^{\pm }$ [51] were calculated by resorting to the HOMO and LUMO energies determined with the MN12SX density functional with results being presented in Table 2. The interested reader in the mathematical formulations of these reactivity descriptors is referred to the original works and to our previous research on the field [17,23,24,25,26,27,28,29].

As expected from the molecular structure of these species, their electrodonating ability is more important than their electroaccepting character as can be seen from the values of the electrodonating and electroaccepting powers and their comparison through the net electrophilicity. However, an interesting comparison can be performed by taking into account the values for the global hardness which is a measure of the deformability of the molecular electron density, and hence, of the chemical reactivity. In this case, it can be observed that deoxoviroidin and deoxoviroisin are much more reactive than the other peptides. This is corroborated by the lower values of the global electrophilicity, i.e., the balance between the chemical electronegativity and the global hardness, for those peptides.

### 3.2. Calculation of the pKas of the Five Fungal Peptides of the Virotoxin Family

In a recent work, a relationship between the pKas of small peptides and the chemical hardness was developed in our group [52] leading to the conclusion that it represents a starting point for the prediction of the pKa of bigger peptides which could be of interest for the development of new therapeutic drugs. The relationship we used in this work for the prediction of the pKas has been already validated within a previous study [52], where it was shown that it can be used to reproduce the experimental pKa of some small peptides.

According to methodology employed in our previous work, we have applied the mentioned relationship of the form pKa = 16.3088 − 0.8268 η to the calculation of the pKa of the fungal peptides considered in this study, with the η values presented in Table 2 being the results as follows.

These results could be of interest for the development of pharmaceutical drugs starting from these molecules enabling at the same time to obtain an explanation about the mechanisms of action and drug delivery procedures. Moreover, they can be shown as an additional application of the results of the calculation of the global reactivity descriptors to the new field of Computational Peptidology [1] and as a possible basis for explaining the solubilities of the peptides.

### 3.3. Local Reactivity Descriptors Calculation

We now turn our attention to the local descriptors of chemical reactivity, namely the Electrophilic Fukui function $f^{-}\left(r\right)$ [9,48,53], the Nucleophilic Fukui function $f^{+}\left(r\right)$ [9,48,53] and the Dual Descriptor (DD) Δf(r) [54,55,56,57,58]. As for the case of the global reactivity descriptors, the interested reader in the mathematical formulations of these reactivity descriptors is referred to the original works and to our previous research on the field [17,23,24,25,26,27,28,29].

**Figure 2:**
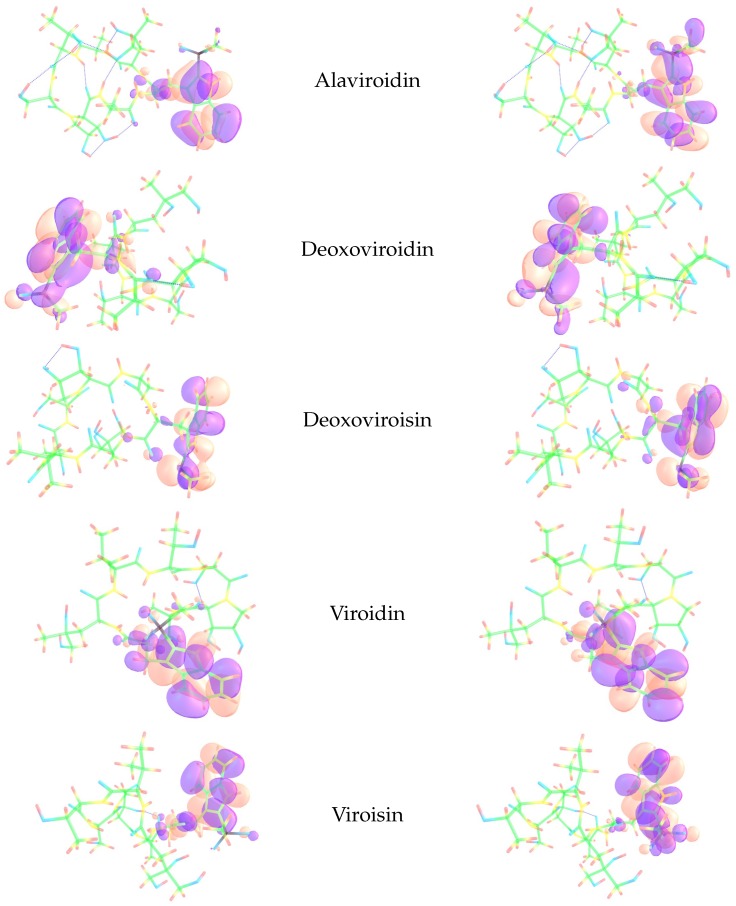
Graphical representation of the Electrophilic Fukui function $f^{-}\left(r\right)$ (left column) and Nucleophilic Fukui function $f^{+}\left(r\right)$ (right column) of the five fungal peptides of the Virotoxin family.

The Electrophilic Fukui functions $f^{-}\left(r\right)$ and Nucleophilic Fukui functions $f^{+}\left(r\right)$ for the five fungal peptides of the Virotoxin family are shown in Figure 2.

Martínez-Araya has explained in recent research [58] that the condensed expression for DD as $\Delta f_{k}$ will be more useful for the prediction of the preferred sites of reaction than the condensed Fukui functions alone. For this reason, we have decided to present the results for the Condensed DD $\Delta f_{k}$ in comparison with the Nucleophilic and Electrophilic Parr functions, $P_{k}^{+}$ and $P_{k}^{-}$, proposed by Domingo et al. [59,60] through the consideration of atomic spin densities that result from a Mulliken Population Analysis (MPA).

The definitions for the Parr functions are [59,60]:
Nucleophilic Parr Function$P{}^{-}\left(r\right)=\rho_{s}^{rc}\left(r\right)$

Electrophilic Parr Function$P{}^{+}\left(r\right)=\rho_{s}^{ra}\left(r\right)$

where $\rho_{s}^{rc}\left(r\right)$ and $\rho_{s}^{ra}(r$) are related to the atomic spin density of the radical cation or anion of the considered system, respectively [16].

The results for the calculation of these local reactivity descriptors for the five fungal peptides of the Virotoxin family are presented in Table 3 where the Condensed Dual Descriptor $\Delta f_{k}$ has been determined by localizing the corresponding Fukui functions over the atomic sites employing a charge scheme based on the MPA as it was done for the Parr functions. It must be noticed that we are presenting only the results for those atomic sites where the $\Delta f_{k}$ are maxima in absolute value. The values for $\Delta f_{k}$ are multiplied by 100 for easier comparison.

As can be seen from Table 4, there is a nice agreement between the results that come from the Condensed Dual Descriptor $\Delta f_{k}$ and those obtained through the Nucleophilic and Electrophilic Parr Functions $P_{k}^{+}$ and $P_{k}^{-}$. Thus, it can be expected that the methodology used in this work could be the basis for the study of the chemical reactivity of therapeutic peptides of larger size. Moreover, by comparing the results from Table 3 and the graphics in Figure 2, it can be concluded that there is a perfect match for both kind of analysis.

## 4. Conclusions

In this work, the chemical reactivity of a group of five members of the Virotoxin family of fungal peptides was studied by resorting to the Conceptual DFT as a tool to explain the molecular interactions.

The information about the global and local reactivity descriptors of the fungal peptides acquired in this work could be helpful in assisting in the design of new pharmaceutical drugs based on these compounds.

Among the many descriptors that could be useful for the development of new medicines, the pKa is of paramount importance because it is related to the water solubility of drugs. Thus, when the experimental values of the pKa are unknown, the approximate QSAR relationship employed in this work could be a useful predictive tool for the determination of the pKas of peptides of similar size as those considered during the development of that expression.

## Figures and Tables

**Table 1:** The HOMO and LUMO orbital energies (in eV), the HOMO-LUMO gap (also in eV) and the maximum absorption wavelengths $\lambda_{max}$ (in nm) of the five peptides of the Virotoxin family predicted by the MN12SX/Def2TZVP/H2O model chemistry.

	HOMO	LUMO	HOMO-LUMO Gap	$\lambda_{\max}$
Alaviroidin	−6.072	−1.700	4.372	284
Deoxoviroidin	−5.929	−1.467	4.462	278
Deoxovirosin	−5.681	−1.228	4.453	278
Viroidin	−6.056	−1.622	4.434	280
Viroisin	−5.888	−1.593	4.295	289

**Table 2:** Global reactivity descriptors of the five members of the Virotoxin family of fungal peptides (in eV), calculated with the MN12SX/Def2TZVP/H20 model chemistry.

**Molecule**	**Electronegativity**	**Global Hardness**	**Electrophilicity**
Alaviroidin	3.886	4.372	1.727
Deoxoviroidin	3.698	4.462	1.533
Deoxoviroisin	3.454	4.453	1.340
Viroidin	3.839	4.434	1.662
Viroisin	3.740	4.295	1.629
**Molecule**	**Electrodonating Power**	**Electroaccepting Power**	**Net Electrophilicity**
Alaviroidin	5.670	1.784	7.454
Deoxoviroidin	5.193	1.495	6.688
Deoxoviroisin	4.685	1.231	5.916
Viroidin	5.520	1.682	7.202
Viroisin	5.396	1.655	7.051

**Table 3:** pKas of the fungal peptides of the Virotoxin family.

Molecule	pKa
FAR	12.69
FAY	12.62
FVY	12.63
FWC	12.64
FWY	12.76

**Table 4:** Local reactivity descriptors for the five fungal peptides of the Virotoxin fmaily calculated with the MN12SX/Def2TZVP/H2O model chemistry: Condensed Dual Descriptor $\Delta f_{k}$, Nucleophilic Parr Function $P_{k}^{+}$ and Electrophilic Parr Function $P_{k}^{-}$.

Alaviroidin			
Atom	$\Delta f_{k}$	$P_{k}^{+}$	$P_{k}^{-}$
50 C	8.71	0.227	0.007
24 N	−13.59	0.048	0.275
**Deoxoviroidin**			
Atom	$\Delta f_{k}$	$P_{k}^{+}$	$P_{k}^{-}$
1 S	6.43	0.071	0.010
48 C	−11.44	0.103	0.358
**Deoxoviroisin**			
Atom	$\Delta f_{k}$	$P_{k}^{+}$	$P_{k}^{-}$
59 C	12.91	0.259	0.067
49 C	−15.01	0.058	0.386
**Viroidin**			
Atom	$\Delta f_{k}$	$P_{k}^{+}$	$P_{k}^{-}$
50 C	7.07	0.268	0.045
24 N	−10.04	0.050	0.225
**Viroisin**			
Atom	$\Delta f_{k}$	$P_{k}^{+}$	$P_{k}^{-}$
1 S	7.83	0.086	0.005
25 N	−9.77	0.044	0.206
